# Supplementary material for: Nrf2 Deficiency Exacerbates the Decline in Swallowing and Respiratory Muscle Mass and Function in Mice with Aspiration Pneumonia
Source: Int J Mol Sci. 2024 Nov 4;25(21):11829. doi: 10.3390/ijms252111829 (PMC11546094; doi:10.3390/ijms252111829)
Supplement: Supplementary file 1 [file ijms-25-11829-s001.zip › ijms-3264048-supplementary.pdf]

The high magnification images of the lung tissue sections

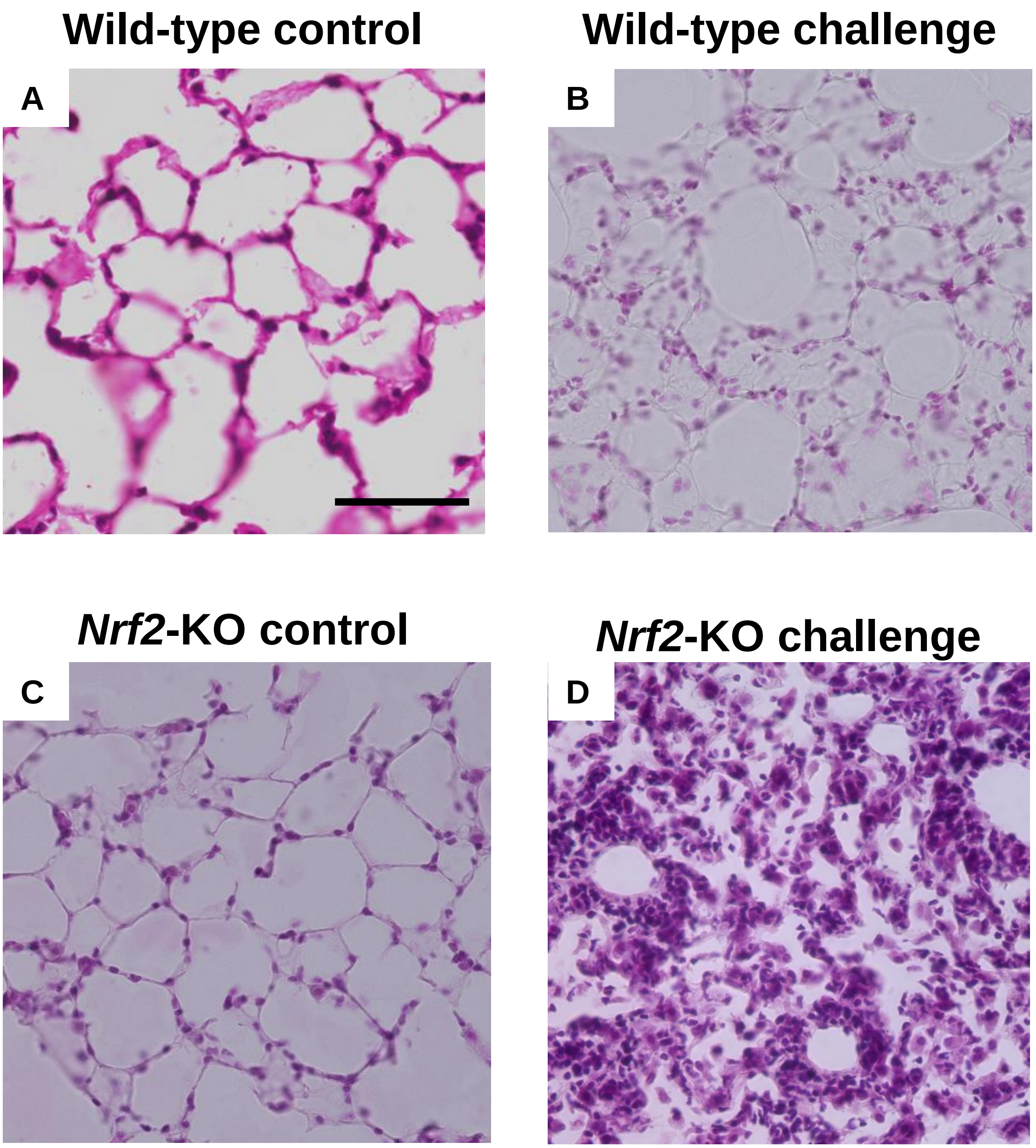

The scale bar in A is 100 µm

Supplemental Figure S1; Hashimoto et al.,

mRNA expression levels of the controls

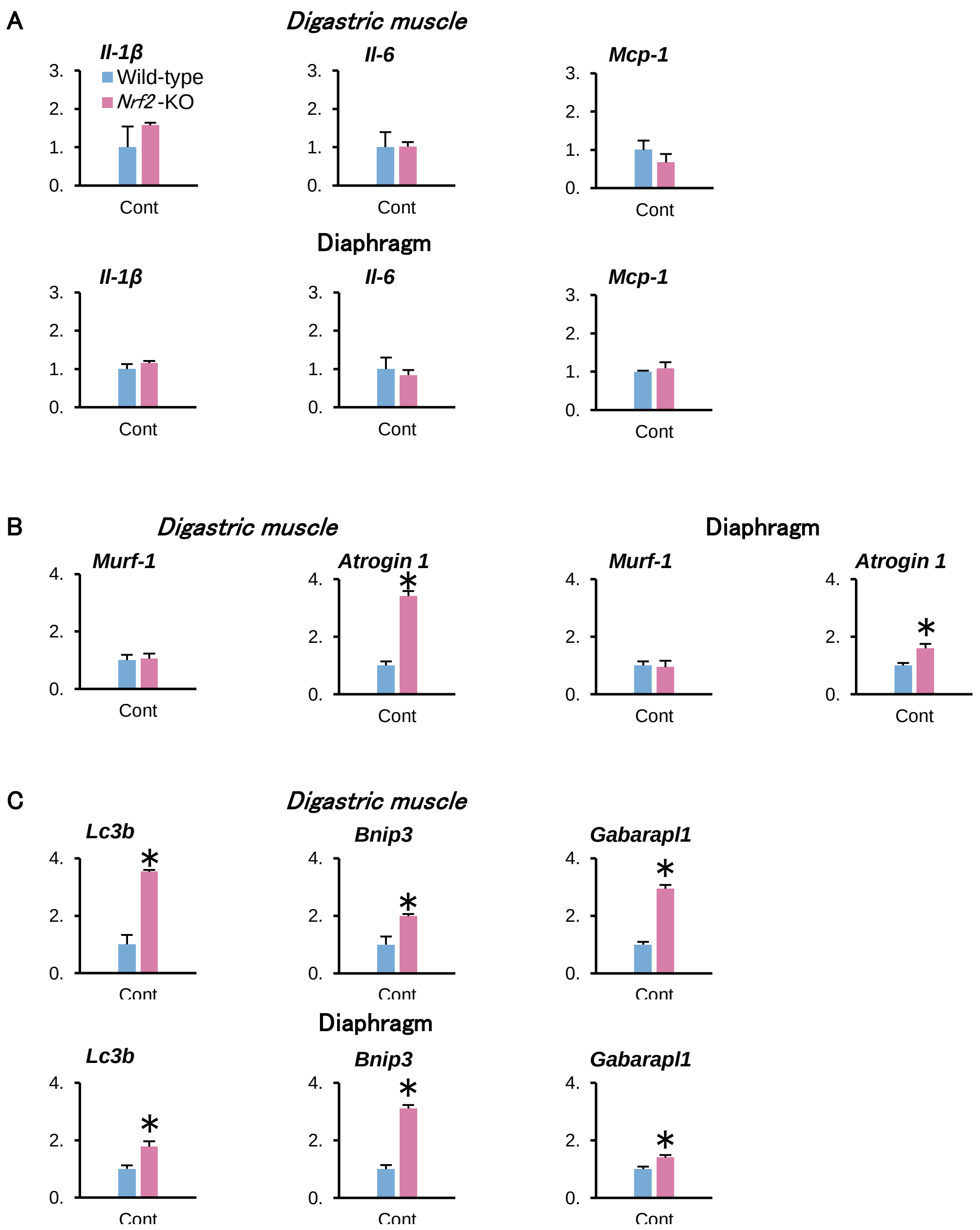

Supplemental Figure S2; Hashimoto et al.,

Comparison of the muscle atrophy within the wild-types and *Nrf2*-knockouts

A *The frequency distribution of the cross-sectional area for Digastric muscle myofibers*

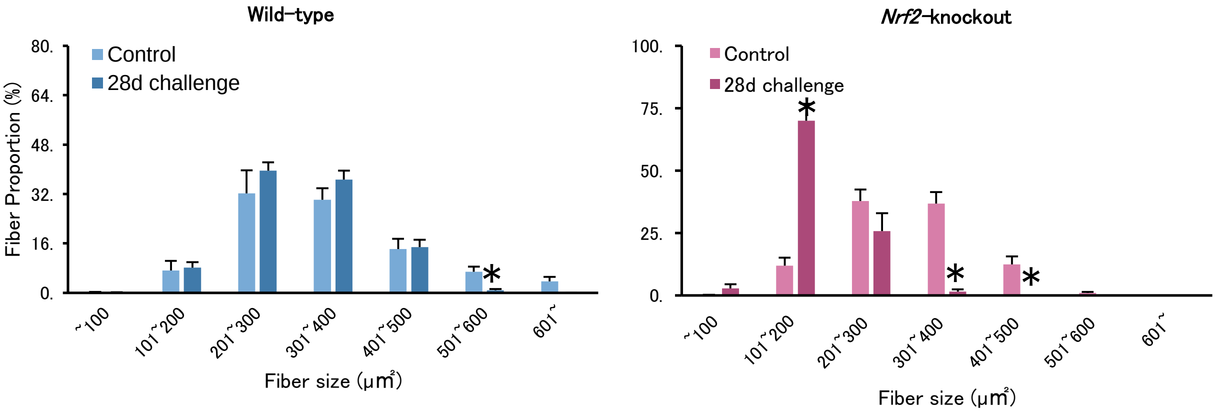

B *The frequency distribution of the cross-sectional area for Diaphragm myofibers*

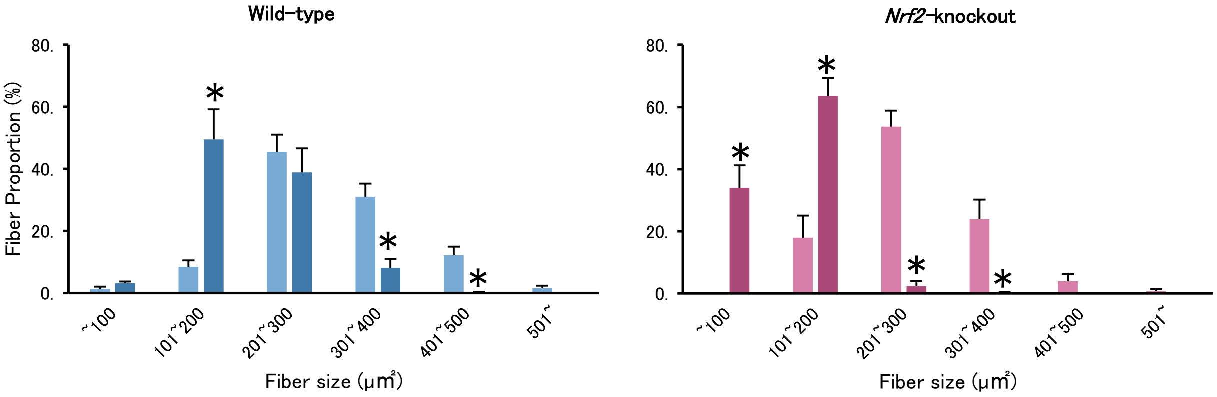

Supplemental Figure S3; Hashimoto et al.,

Primer sequences

Gene target	Forward primer	Reverse primer
Il-1β	5’-GGGCCTCAAAGGAAAGAATC-3’	5’-TACCAGTTGGGGAACTCTGC-3’
Il-6	5’-CCGGAGAGGAGACTTCACAG-3’	5’-CAGAATTGCCATTGCACAAC-3’
Mcp-1	5’-CCCAATGAGTAGGCTGGAGA-3’	5’-TCTGGACCCATTCCTTCTTG-3’
Murf-1	5’-ACCTGCTGGTGGAAAACATC-3’	5’-AGGAGCAAGTAGGCACCTCA-3’
Atrogin-1	5’-ATTCTACACTGGCAGCAGCA-3’	5’-TCAGCCTCTGCATGATGTTC-3’
Bnip3	5’-TTCCACTAGCACCTTCTGATGA-3’	5’-GAACACCGCATTTACAGAACAA-3’
Lc3b	5’-CCGGAGCTTTGAACAAAGAGTG-3’	5’-CTTGGTCTTGTCCAGGACGG-3’
Gabarapl1	5’-CAGCTGTATGAGGACAACCAC-3’	5’-CAAGTCCAGGTGCTCCCAT-3’
β-actin	5’-CGACAACGGCTCCGGCATGT-3’	5’-TCTGGGCCTCGTCACCCACA-3’

Supplemental Table S1; Hashimoto et al.,

Product code of the primary antibodies

Primary antibody	Concentration	Product code	Company
S100 calcium-binding protein A8	1:500	AF3059	R&D Systems, Minneapolis, MN
F4/80	1:100	MCA497G	Bio-Rad, Hercules, CA
CD3e	1:500	eBio500A2	eBioscience, San Diego, CA
B220/CD45R	1:500	RA3-6B2	eBioscience, San Diego, CA
Anti-α-fodrin	1:1000	BML-FG6090-0100	ENZO, New York, NY
Anti-p62	1:1000	PM066	MBL, Tokyo, Japan
Anti-α Tubulin	1:2000	T9026-100UL	Sigma-Aldrich, Saint Louis, MO

Supplemental Table S2; Hashimoto et al.,
